# Higher Participation Rate for Specific Health Checkups Concerning Simultaneous Ophthalmic Checkups

**DOI:** 10.2188/jea.JE20200052

**Published:** 2021-05-05

**Authors:** Yoshimune Hiratsuka, Tetsuji Yokoyama, Masakazu Yamada

**Affiliations:** 1 Department of Ophthalmology, Juntendo University School of Medicine, Tokyo, Japan; 2 Department of Health Promotion, National Institute of Public Health, Saitama, Japan; 3 Kyorin Eye Center, Kyorin University, Tokyo, Japan

**Keywords:** ophthalmic checkup, participation rate for specific health checkups, specific health checkups, eye checkup, visual impairment

## Abstract

**Background:**

Improving the specific health checkups participation rate is an essential task nationwide; however, studies on measures to accomplish this are limited. This study aimed to examine the influence of ophthalmic checkups on the specific health checkups’ participation rate.

**Methods:**

We conducted a postal questionnaire survey on 1,741 countrywide municipalities in Japan from January to February 2019. The questionnaire specifically addressed health checkup participation rates of 2017, health checkups formats (group, individual, or both), simultaneous cancer screenings, simultaneous ophthalmic checkups, and the state of implementation of ophthalmic checkups. We used multiple linear regression analyses to calculate the partial regression coefficients (βs) and their 95% confidential intervals (CIs) to identify the influence of simultaneous ophthalmic checkups on specific health checkup participation rates.

**Results:**

There was a significant association between specific health checkup participation rates and simultaneous ophthalmic checkups (β: +2.5%; 95% CI, 1.3–3.8) after adjusting for covariates. The fundus photos of all applicants, fundus photos with restrictions, and ophthalmology consultation for all applicants were associated with a significant increase in the specific health checkup participation rate (β: +2.8%, 95% CI, 1.2–4.4; β: +2.0%, 95% CI, 0.2–3.9; β: +7.4%, 95% CI, 1.2–13.6 respectively).

**Conclusions:**

Our results suggest that additional simultaneous ophthalmic checkups as specific health checkups could increase the specific health checkup participation rate.

## INTRODUCTION

“Specific Health Checkups” is an annual health screening program introduced by the Japanese Ministry of Health, Labour and Welfare in 2008. The aim of the program is the early detection of individuals at high risk of metabolic syndrome to enable healthcare intervention. Currently, the national participation rate for specific health checkups is 53.1%; however, the participation rate of individuals covered by health insurance unions is 77.3% and that of individuals covered by municipalities (municipal national health insurance) is 37.2%.^1^ Therefore, improving the participation rate for specific health checkups is an essential task for municipalities nationwide. There are also big differences in the participation rates among prefectures, from 40.4% in Hokkaido to 64.8% in Tokyo, for example.^2^ Local governments are implementing various strategies to improve the participation rate of specific health checkups. These include conducting consultation recommendations (eg, telephone recommendations and consultation recommendation leaflets) for unscreened persons, expanding specific health checkup consultation locations, and providing information services.

An ophthalmic checkup is a screening aimed at detecting eye diseases early in the general population. The fundus examination is the most common method of performing an ophthalmic checkup. Signs of hypertensive retinopathy—which are identifiable by a fundus examination—are predictive of incident stroke, congestive heart failure, and cardiovascular mortality, independent of traditional risk factors.^3^

Fundus examinations in the health checkup system used to be conducted based on the judgment of the doctor concerned; however, the implementation criteria have changed significantly since the introduction of specific health checkups and a specific health guidance scheme in 2008. Specifically, after meeting certain criteria as a “detailed medical examination item” based on metabolic risk factors, it is implemented only when the doctor decides that it is necessary. As a result, the rate of fundus examinations decreased significantly.^4^ There are, however, municipalities that include ophthalmologic examinations apart from their “detailed medical examination items” in specific health checkups efforts covered by health insurance. For example, in Matsue City, Shimane, Setagaya Ward, Tokyo, and Sendai City, Miyagi, all health insurance candidates are eligible for an ophthalmologic examination at the same time of the specific health checkup examinations, whether or not they comply to certain criteria or not. However, even if these ophthalmologic examinations on insured individuals are carried out, the participation rate of ophthalmic checkups in relation to specific health checkups is only 13.4%.^5^

Although providing cancer screening at the same time of specific medical checkups is suggested to improving the participation rate,^6^ it is still unclear what influence simultaneous ophthalmic checkups has on specific health checkup participation rates. Therefore, this study conducted a questionnaire survey on municipalities across the country to examine the influence of ophthalmic checkups on specific health checkups’ participation rate.

## METHODS

### Participants and dataset

The target population comprised of all 1,741 municipalities across the country in Japan. We conducted a questionnaire survey by mail aimed at regional health and health promotion personnel in each municipality from January to February 2019. The questionnaire contents addressed the specific health checkup participation rate of 2017, health checkup formats, simultaneous cancer screenings, simultaneous ophthalmic checkups, and the state of implementation of ophthalmic checkups. Health checkup formats were divided into three groups: group, individual, and both. The group health checkups are conducted at health centers in various parts of municipalities. The individual health checkups are performed by consulting a specific medical institution. “Both” implement both group and individual health checkups. Returning the completed questionnaire was considered as informed consent to participation.

### Statistical analysis

All study variables were added to our descriptive analysis. We used a *t*-test to compare the difference of the specific health checkup participation rate in municipalities of similar population sizes, whether conducting simultaneous ophthalmic checkups. We then performed a multiple linear regression analysis to calculate the partial regression coefficients (βs) and their 95% confidential intervals (CIs) to identify the influence of simultaneous ophthalmic checkups on specific health checkup participation rates. We adjusted for the following possible confounding factors: specific health checkups formats (group, individual, or both), simultaneous cancer screening, prefecture (47 prefectures), and municipal population size (≥1,000,000, ≥500,000, ≥300,000, ≥100,000, ≥50,000, ≥10,000, and <10,000). Next, a multiple linear regression analysis was performed to observe the effects of the procedure included in ophthalmic checkups (fundus photos for all applicants, fundus photos with restrictions, ophthalmology consultation for all applicants, ophthalmology consultation with restrictions, and other). We used Stata 14 software (Stata Corp., College Station, TX, USA) for the analyses and set the significance level at 5%.

### Ethical considerations

Ethical approval for this study was obtained from the ethics committee of Kyorin University, approval number: H30-128.

## RESULTS

Of the original 1,741 municipalities, 1,075 responded to the questionnaire (response rate: 61.7%). Responses were obtained from municipalities belonging to all 47 prefectures. Of the 1,075 respondents, the final analysis included 1,048 municipalities, with 27 municipalities being excluded due to information on key variables (specific health checkups participation rate, simultaneous ophthalmic checkups, specific health checkup format, and simultaneous cancer screening) being incomplete.

The mean specific health checkup participation rate was 41.4% (standard deviation, 10.3). Concerning specific health checkup format, 76% of the specific health checkups conducted by municipalities were in both group health checkup and individualized health checkup format. The percentage of municipalities offering only group health checkups was 14%. The proportion of municipalities that offer simultaneous cancer screenings with specific health checkups was as high at 92%. Three hundred and ten municipalities (30%) offer ophthalmic checkups individually—as opposed to included in “detailed medical examination”—in specific health checkups. The demographic characteristics of municipalities are shown in Table 1. The crude analysis comparing the specific health checkup participation rate among municipalities categorized by the population size was higher in municipalities with ophthalmic checkups in all population size groups. However, in municipalities of lower population size (“50,000 to 100,000” and “<10,000”), the difference was not statistically significant (Table 2).

**Table 1.** Data of study variables from questionnaire

	Municipalities
	(*N* = 1,048)
Consultation rate for specific health checkups, mean (SD)	41.4 (10.3)

Specific health checkups formats, number (%)	
Group health checkup	140 (13.4)
Individualized health checkup	108 (10.3)
Both group and individual	800 (76.3)

Cancer screening, number (%)	
Yes	964 (91.9)
No	84 (8.0)

Ophthalmic checkup examination, number (%)	
Yes	310 (29.6)
No	738 (70.4)
Ophthalmic checkup examination procedure, number (%)	
Fundus photos for all applicants	179 (17.1)
Fundus photos with restrictions	111 (10.6)
Ophthalmology consultation for all applicants	8 (0.8)
Ophthalmology consultation with restrictions	6 (0.6)
Others	6 (0.6)
Population size, number (%)	
≥1 million	9 (0.9)
500,000 to 1 million	18 (1.7)
300,000 to 500,000	39 (3.7)
100,000 to 300,000	143 (13.7)
50,000 to 100,000	180 (17.2)
10,000 to 50,000	418 (39.9)
<10,000	241 (23.0)

**Table 2.** The specific health checkup participation rate among municipalities categorized by the population size with or without ophthalmic checkups

Ophthalmic checkup examination					

Population size	Yes		No		*P* value
	*n*	Participation rate (%)	*n*	Participation rate (%)	
≥1 million	2	44.0	7	27.3	0.01
500,000 to 1 million	7	42.3	11	32.9	0.01
300,000 to 500,000	7	43.5	32	37.2	0.01
100,000 to 300,000	37	39.9	106	37.1	0.04
50,000 to 100,000	49	40.5	131	39.7	0.49
10,000 to 50,000	107	43.3	311	40.9	0.03
<10,000	91	46.7	150	44.9	0.27

The factors associated with the specific health checkup participation rate were analyzed using multiple linear regression analyses (Table 3). The results showed a significant association between the specific health checkup participation rate and simultaneous ophthalmic checkups (β, +2.5%; 95% CI, 1.3–3.8) after adjusting for other confounders (Adjusted R-squared = 0.30). Moreover, simultaneous cancer screening and the specific health checkup formats were not significantly associated with the specific health checkup participation rate. Table 4 shows the results of the additional analysis concerning the procedures of the ophthalmic checkup. The fundus photos for all applicants with restrictions and the ophthalmology consultation for all applicants were associated with a significant increase in the specific health checkup participation rate (β: +2.8%, 95% CI, 1.2–4.4; β: +2.0%, 95% CI, 0.2–3.9; β: +7.4%, 95% CI, 1.2–13.6 respectively) (Adjusted R-squared = 0.30).

**Table 3.** The factors associated with the specific health checkup participation rate using multiple linear regression analyses

	Partial regression coefficient	*P* value	95% CI
**Ophthalmic checkup examination**			
Yes/No	2.5	<0.001	(1.3, 3.8)

Cancer screening			
Yes/No	−0.1	0.96	(−2.2, 2.1)

Specific health checkups format			
Group health checkup	reference		
Individualized health checkup	2.6	0.06	(−0.1, 5.3)
Both group and individual	−0.7	0.42	(−2.5, 1.0)

Population size			
≥1 million	reference		
500,000 to 1 million	1.8	0.62	(−5.3, 8.9)
300,000 to 500,000	3.8	0.24	(−2.6, 10.2)
100,000 to 300,000	4.5	0.13	(−1.4, 10.5)
50,000 to 100,000	6.0	0.05	(0.1, 11.9)
10,000 to 50,000	8.5	0.004	(2.7, 14.3)
<10,000	12.8	<0.001	(6.9, 18.7)

CI, confidence interval.

All values are adjusted for 47 prefectures.

**Table 4.** The association between the specific health checkup participation rate and the procedures of the ophthalmic checkup

Ophthalmic checkup examination	Partial regression coefficient	*P* value	95% CI
No	reference		
Yes			
Fundus photos for all applicants	2.8	<0.001	(1.2, 4.4)
Fundus photos with restrictions	2.0	0.03	(0.2, 3.9)
Ophthalmology consultation for ​ all applicants	7.4	0.02	(1.2, 13.6)
Ophthalmology consultation with ​ restrictions	−1.7	0.64	(−9.0, 5.5)
Others	1.2	0.74	(−6.0, 8.5)

CI, confidence interval.

All values are adjusted for other confounders in Table 3.

## DISCUSSION

The results of this study suggested that additional simultaneous ophthalmic checkups as part of specific health checkups could increase the specific health checkup participation rate.

Approximately 10 years have passed since the implementation of the specific health checkups program in Japan, and the participation rate—38.9% in 2008—has improved to 53.1% in 2017.^1^ Despite steady improvement, it is still far from the target rate of 70% set by the government, and efforts are needed to further improve the participation rate. Considering the health checkup rate of specific health checkup providers, the rates are (in descending order) 78% for mutual aid associations (85 insurers), 77% for health insurance associations (1,385 insurers), 49% for Japan health insurance association (1 insurer), 49% for national health insurance association (163 insurers), 37% for municipal national health insurance (1,738 insurers), and 36% for seamen’s insurance (1 insurer).^1^ From the above, the most pressing problem is the low participation rate of individuals covered by municipalities (municipal national health insurance), approximately 16% of the total population.

The factors associated with increasing the health examination participation rate have been discussed in existing literature. Positive factors for participation include being active in social networks,^7^^,^^8^ and socioeconomic factors such as high educational attainment, house ownership, and low equivalent household expenditure.^9^ However, little is known about the possible interventions to increase participation in health checkups.

Municipal national health insurance and the national health insurance association, as well as other insurers, are using various approaches to improve participation. For example, they conduct specific health checkups and cancer screenings at the same time, promote lifestyle-related disease prevention medical examinations, and collaborate with family medical institutions. Moreover, attempts are being made to secure medical examination opportunities that are available at night and on holidays, to provide easy-to-access venues, and to recommend consultations at the right time, according to the health checkup schedule.^6^ As an example, it has been shown that the participation rate improved from 11.5% to 24.1% in 2 years due to offering skin age and bone density measurements at the same time as specific health checkups and by offering a medical examination venues close to living areas.^6^ Our study showed that there was no significant association between the specific health checkup participation rate and simultaneous cancer screening. The simultaneous cancer screening may already have been sufficiently high (92%) that no significant effects were observed.

Although existing literature proposes other screening options as an attractive solution for improving the participation rate of health checkups, there has been no mention of ophthalmic checkups. As stated before, it is possible to contribute to improving the participation rate for specific health checkups by conducting other independent checkup examinations—such as ophthalmic checkups—at the same time as specific health checkups.

There are several ophthalmic screening procedures that are currently being implemented. The most common procedure is taking fundus photographs of all applicants. There are also municipalities that take fundus photos with some restrictions, such as age, first-come-first-served, and a paid procedure for those who wish it. Moreover, there are some local governments that allow all applicants to have ophthalmology consultations. Interestingly, in our analyses, both unrestricted ophthalmic checkups (fundus photos for all applicants; +2.8% or ophthalmology consultation for all applicants; +7.4%) were significantly associated with an increase in the participation rate for specific health checkups. These results suggest that it is more likely for a simultaneous health check without any restrictions to be effective in improving the participation rate for specific health checkups than a health check with restrictions on participation.

Ophthalmic screenings are cost effective and can detect not only abnormalities due to high blood pressure and diabetes,^10^ but also many other eye diseases, such as glaucoma^11^ and age-related macular degeneration.^12^ More than 90% of people with glaucoma—currently the leading cause of blindness in Japan—are undiagnosed.^13^ Concerning diabetic retinopathy, for which annual fundus examinations are recommended,^14^ the annual ophthalmic visit rate is approximately 36%.^15^ Therefore, setting such examinations as options for specific medical checkups is useful not only for improving the participation rate, but also for diagnosing undiscovered eye diseases. Visual impairment is a significant contributor to a country’s burden of disease and the social cost of visual impairment is proportionally higher in super-aging societies, such as Japan.^16^ In Japan’s national health promotion plan in the decade from 2013, the extension of healthy life expectancy (HLE) is a key factor. HLE is the period that an individual can live with full health or perfect health. Therefore, decreasing the difference between lifespan and HLE is presently an important task in Japan. To extend HLE, it is necessary to shorten the period of decreased independence and requiring assistance or long-term care. Some of the main reasons for needing for long-term care in 2016 were as follows: dementia, frailty, stroke, falls/fractures, arthritic disorders, heart disease, diabetes, and visual and hearing impairment.^17^ Most of these are closely related to visual impairment. For example, it has been said that people with visual impairment have lower cognitive levels than those without visual impairment.^18^ Vision-related quality of life and poor visual acuity are also significantly associated with physical inactivity.^19^ Moreover, it has been stated that fundus examinations can contribute to the prevention of stroke or the development of heart disease.^3^ Lastly, visual impairment is also associated with increased risk of falls.^20^^,^^21^ Taken together, these factors show that there is a strong association between visual impairment and reasons for requiring long-term care or support. Moreover, population attributable fractions of activity limitation—which refers to the proportion of people for whom activity limitation would be expected to decrease if no participant suffered from the disease—is second highest in ophthalmic diseases, following orthopedic diseases, among 38 diseases in Japan.^22^ All of the above shows that measures against visual impairment will help to extend HLE, confirming that there are many advantages to regular eye examinations.

One of the greatest strengths of this study is its large-scale sample of nationwide municipalities. Another advantage is that this is the first study to investigate the influence of independent simultaneous checkups on the specific health checkups’ participation rate.

It should be noted that our study had several limitations. First, the response rate was moderate (61.7%); therefore, our results may have been affected by response bias. Additionally, the response rate was lower in municipalities with a population size of fewer than 10 thousand (47.3%); hence, our results may not be generalizable to a municipality with a small population. Second, the target of this study is municipalities (municipal national health insurance), and the participation rate (37%) is much lower than that of other health insurance providers, such as health insurance associations (77%). Therefore, it may not be applicable to providers who already have a high participation rates. Third, our study followed a cross-sectional design; therefore, it was not possible to generate any causation results. The present cross-sectional study could not exclude the possibility of reverse causation, in that municipalities with a high participation rate for specific health checkups tend to provide simultaneous ophthalmic checkup to the residents. Longitudinal studies or intervention studies are necessary to examine the effects of simultaneous ophthalmic checkup on participation rate for specific health checkups. Lastly, as with all survey data, responses may have been subject to recall bias and some respondents may have answered certain questions inappropriately. However, the respondents in this study were regional health and health promotion personnel and therefore, it can be reasonably assumed that reliable answers were obtained.

In conclusion, offering additional simultaneous ophthalmic checkups as part of specific health checkups could increase the specific health checkup participation rate. Setting such examinations as options for specific medical checkups could be useful, not only for improving the participation rate, but also for diagnosing undiscovered eye diseases. This may, in turn, lead to extended HLE in the general population.
